# A Holistic Approach to Early Relational Health: Cultivating Culture, Diversity, and Equity

**DOI:** 10.3390/ijerph21050563

**Published:** 2024-04-28

**Authors:** Dominique Charlot-Swilley, Kandace Thomas, Christina F. Mondi, David W. Willis, Marie-Celeste Condon

**Affiliations:** 1 Department of Pediatrics, Georgetown University School of Medicine, Washington, DC 20007, USA; 2 First 8 Memphis, Memphis, TN 38104, USA; ksrthomas@gmail.com; 3 Brazelton Touchpoints Center, Division of Developmental Medicine, Boston Children’s Hospital, Harvard Medical School, Boston, MA 02115, USA; christina.mondi-rago@childrens.harvard.edu; 4 Center for the Study of Social Policy, Washington, DC 20005, USA; dwwillis1950@gmail.com; 5 Independent Consultant and Researcher, University of Washington, Seattle, WA 98105, USA

**Keywords:** relational health, infancy, Indigenous, cultural contexts, diversity, equity, village wellbeing

## Abstract

Early Relational Health (ERH) is the foundation for infant and child emotional and social wellbeing. ERH is a quality of relationships co-created by infants, caregivers, and other members of their families and communities from pregnancy through childhood. Relationships themselves are not ERH; rather, ERH can be a feature of relationships. Those that are characterized by positive, shared emotionality become contexts within which members co-develop mutual capacities that enable them to prevail and flourish. This essay offers a synthesis of current knowledge about ERH in the US and begins to integrate Indigenous and non-Indigenous research and knowledge about ERH in the hope that readers will embrace “*Etuaptmumk*”—“*Two-Eyed Seeing*”. The authors maintain that systems of care for infants, families, and their communities must first and foremost attend to revitalization, cultural context, diversity, equity, and inclusion. Authors discuss key concepts in ERH; Indigenous and non-Indigenous research that inform ERH; structural and systemic factors in the US that affect ERH ecosystems; the critical intersections of culture, diversity, equity; the broader concept of village support for fostering ERH; and efforts to revitalize ERH discourse, practices, and policies. The authors advocate for a holistic approach to ERH and suggest future directions for research and advocacy.

## 1. Introduction

Early Relational Health (ERH) is informed by research about the influence of positive, shared emotional experiences and nurturing relationships during the early years of life [1,2]. ERH is a quality of relationships co-created by infants, caregivers, and other members of their families and communities from pregnancy through childhood. Relationships themselves are not ERH; rather, ERH can be a feature of relationships. Those that are characterized by positive, shared emotionality become contexts within which members co-develop mutual capacities that enable them to prevail and flourish [2,3,4,5], for example, mutual capacities like feeling calm in one another’s presence, being interested in connecting, and able to feel delighted in and delightful to one another [6]. The naissance of relationship and the possibility of ERH happen when birthing and adoptive parents begin bonding with the infant and envisioning a hopeful future together [7]. 

The aims of this paper are to offer a synthesis of current knowledge about ERH in the US; begin to integrate Indigenous and non-Indigenous research and knowledge about ERH in the hope that readers will embrace “*Etuaptmumk*”—“*Two-Eyed Seeing*” [8,9]; and contribute to international dialogue about holistic infant–family ERH care. The authors maintain that systems of care for infants, families, and their communities must first and foremost attend to revitalization, cultural context, diversity, equity, and inclusion. In this vein, providers are called to decolonize and revitalize their thinking and engage in holistic practice with cultural humility. A complex concept without a precise definition, holistic care has been referred to as “the heart of the science of nursing” [10,11] (p. 91). For the purposes of this paper, a holistic approach is based on a mutual understanding of infants, families, and communities’ cultural contexts, strengths, needs, perspectives, and factors that impact their ERH. It is wise to begin relationships with optimism, inclusivity, mutuality, and parallel process in mind, so communities and families feel seen, heard, understood, and a sense of authentic invitation to co-create safe, stable, and nurturing relationships (SSNR) [12] that are a major objective of ERH.

In this paper, we explain key concepts in ERH; present and constructively critique studies by Indigenous and non-Indigenous researchers that inform and support ERH; speak to structural and systemic factors in the US that affect ERH ecosystems; offer a few exemplars of efforts to revitalize ERH discourse and practices; and discuss future directions. 

## 2. Key Concepts in ERH 

ERH appears to be a universal phenomenon, in that all around the world, infants and the people who care for them clearly connect with one another for the survival of the infant and the benefit of all. As adults prepare for parenthood, important neurobiological changes take place [13,14,15,16] that help them recognize and respond to their infants’ cues and heighten their attention to infants’ distress. These changes link to nurturing behaviors such as holding, comforting, and mutual connection [17,18,19,20,21,22,23].

“Babies offer a fundamental lesson in the power of not knowing. They enter the world with a unique self, capable of complex communication. Listening to the baby from a stance of flexibility and curiosity, not always “getting” what the baby is communicating but taking the time to figure things out when they are off the mark, caregivers pave the way for social adaptation and the tolerant uncertainty inherent in all social interaction. The ability to find our way into another person’s experience begins to develop when caregivers naturally respond to an infant’s wordless communication. This early experience of being seen and heard builds a primary feeling of connection and belonging [24]…” that is necessary for the naissance of ERH.

Mutual capacities develop in the context of overarching positive emotionality in relationships [6,18,21,22]. This does not mean there are never moments of distress. It simply means healthy relationships are able to manage stress because members have accumulated many shared experiences of successfully joining, engaging in emotional co-regulation and recovering from dysregulation. Mutual capacities in relationships are co-created during synchronous, mutually responsive interactions that enrich the wellbeing of everyone in the relationship.

Although it is common to think in terms of the age of the child, it is also important to consider the age of relationships. Time and the accumulation of positive shared experiences strengthen connections between members of relationships and open the door to the development of mutual capacities [6]. Table 1 shows manifestations of ERH that racially and culturally diverse families, clinicians, and researchers in the US recognize in dyadic and multi-member relationships with infants from the last trimester in the womb through the infants’ fourth birthday. Cultural fluency, culture humility [23,24,25], and compassionate curiosity about participants’ thoughts and feelings, the meanings they understand and convey, and what they seem to cherish or avoid matter when witnessing and making meaning of interactions. Table 1 shows indicators of ERH (mutual capacities) that develop when overarching emotionality in relationships javascript:void(0) is positive.

The examples in Table 1 are not exhaustive or necessarily seen across all cultures or in every relationship [6]. Infants have different meanings for families in different cultures, and consequently, perceptions of family–baby interactions differ across cultures [26,27,28,29,30,31]. Indicators of ERH, such as calm interest in one another, mutual responsiveness, and enjoyment, likely exist across cultures, but the ways in which they are expressed may differ [6]. 

It is as important for members of a culture to discern, reflect upon, and discuss signs of struggle and vulnerability in relationships as it is to discern and discuss signs of vigor. In the US, some indicators of early relational vulnerability that racially and culturally diverse families, clinicians, and researchers have recognized include emotionality that one or more people in the relationship experience as uncertain, distressing, demoralizing, or even threatening. There may be unclear, aggressive, avoidant, or withdrawn behaviors, such as flat or listless facial expressions; crying, grimaces, frowns, glaring, scowling, turning away, yelling, harsh, sarcastic, or critical tones of voice; rough touch or handling; extending arms or fists as if to hit, or extending feet as if to kick. Behaviors like these dysregulate and dishearten relationships during infancy. They are seen, heard, and understood by others in the culture as signs of difficulty between members of a relationship [6,32]. 

In the US, most people think of relationships in dyadic terms, and capacities as individual accomplishments. In fact, relationships between caregivers and infants often encompass three or more members. In many cultures, belonging and contributing to collective wellbeing matter more than individual achievement [33,34]. In places where community and family ties are strong, children have opportunities to develop attachments with multiple caregivers and rely on broader social networks for support [4,5,33,34,35,36,37,38]. The possibility of ERH extends beyond the close relationships they have with family, kin, and other caregivers during the first year of life to multi-member relationships with other members of their village [34,36,38,39]. 

## 3. Evolution of ERH Concepts and Practices in the US

The *Behavioral Health Screen* (BHS) is an instrument for assessing dyadic interactional quality between parents and infants who are 6–24 months of age. It addresses the dyad as a unit, not the child’s or parent’s individual contribution. Parent–child interactions are video-recorded and rated on a 3-point scale (2—clearly observed, 1—sometimes observed, 0—not observed) [40]. The sum score is used, reflecting an overall measure of dyadic interactional quality. The BHS evolved to become the *Early Relational Health Screen* (ERHS) [41]. The ERHS was validated during a population-level Norwegian study [40] and two US studies in primary care and home visiting settings [42]. The BHS and ERHS were developed and validated by White Euro-American researchers with relatively homogeneous samples. The ERHS is useful in research and clinical settings when rating is completed by observers who are fluent in participants’ languages and cultural contexts. Multi-member relationships and the effects of cultural context, implicit bias, and systemic racism on reliability and acceptability have not been investigated. 

A pivot in thinking about relationships with infants occurred during a qualitative, participatory action study of racially and culturally diverse infants’ relational experiences in a residential parenting program in the US [32,43]. Findings revealed evidence of mutuality in capacities in both dyadic and multi-member relationships. When parents and caregivers were invited to share stories about their relationships with infants and reflect on thoughts and feelings they have about their relationship, they become more attuned to and curious about their infants’ thoughts and feelings, and positive shifts in the overarching emotionality of their relationships were clearly seen and felt [32]. These findings were replicated in a participatory action study of the experiences and opinions of racially, culturally, and linguistically diverse families of infants as they witnessed and reflected on videos of their interactions. Families enthusiastically endorsed using their phones to make, watch, and reflect on videos with home visitors about changes in their infant–family relationships over time. Seeing and celebrating signs of flourishing in relationships despite adversity inspired hope and resilience [41].

In a *HealthySteps* [44] pediatric primary care setting, a phenomenological study of the experiences and opinions of African American families and clinicians of Color with ERH-focused visits. Clinicians became increasingly cognizant of the importance of decolonizing (*revitalizing*) ways of “being with” with families [45,46]. The relevance, acceptance, and utility of ERH-focused visits for families was influenced by clinicians’ attention to positionality, cultural context, issues of equity, and how they invited family members to engage in mutual reflection with them. A family-centered approach that framed interactions as narratives, opened space and time for family reflection and access to culturally relevant knowledge about ERH, and affirmed joy made positive differences in engagement and the relevance of ERH-focused visits for families [45]. Results led to the development of a family-reflection and culture-centered model for ERH promotion and intervention called *Early Relational Health Conversations* (ERH-C) [46]. This study represents a pivot from risk- and deficit-focused care toward holistic care and “a hopeful, inclusive, and strengths-focused framework” [2] (p. 14). The model provides concrete guidance about ways to address implicit bias and disparities in family–provider relationships; joining with diverse families and communities; pivoting from hustle to flow [47]; and facilitating dialogue, mutual reflection, and insight to address families and communities’ relational health (RH) concerns from pregnancy through early childhood [48]. 

ERH concepts are capturing the attention of doulas (midwives), infant and early childhood educators, social workers, pediatricians, scientists, and policy makers thanks to the CSSP’s commitment to advancing ERH and its development of *Nurturing Connections*, a partnership with families and communities dedicated to building a networked and engaged movement to promote ERH for every family in every community [49]. *Health Connect One* community-based doulas who live in the same neighborhoods and cultural contexts as the families they serve are adapting and scaling up ERH-C in communities in 27 states in the US [50]. Doulas and families observe, reflect on, and organize referrals around all three facets of ERH: (a) overarching affect within relationships, (b) observable patterns of relationships, and (c) thoughts and feelings individuals have about their relationships with infants and young children [32]. 

## 4. Indigenous and Non-Indigenous Studies and Perspectives

### 4.1. Two-Eyed Seeing: Integrating Knowledge Systems

Non-Indigenous researchers and service providers have much to learn from listening to stories of Indigenous scientists and community members about their experiences of obstacles to and facilitators of relational healing, and ways of being in relationship with one another and infants that enable all to flourish. “*Etuaptmumk”*—"Two-Eyed Seeing” is a way to honor worldviews where physical, emotional, relational, and spiritual needs are of equal importance. Tending to and strengthening broken bonds is a good start to bridging worldviews [51] about RH and ways of nurturing the naissance, development, and sustenance of ERH in different cultural contexts.

“Two-Eyed Seeing”, a guiding principle brought into the Institute for Integrative Science and Health (IISH) by Mi’kmaw Elder Albert Marshall [8], is relevant to ERH work with diverse people. He taught the following:

“*Etuaptmumk*” is the Mi’kmaw word for “Two-Eyed Seeing.” We often explain “*Etuaptmumk”*—"Two-Eyed Seeing” by saying it refers to learning to see from one eye with the strengths of Indigenous knowledges and ways of knowing and from the other eye with the strengths of Western knowledges and ways of knowing… and learning to use both these eyes together, for the benefit of all. “*Etuaptmumk*” is the gift of multiple perspectives treasured by many Aboriginal Peoples. We are also exploring “story as relationship.”

More can be learned by visiting IISH’s website at http://www.integrativescience.ca/Principles/ (accessed on 20 March 2024) [52].

“*Etuaptmumk”*—“Two-Eyed Seeing” is a way to bridge and integrate non-dominant and dominant knowledge systems; conduct trustworthy community-based participatory action studies; repair environments and relationships; and leave relationships and the world a better place for the next seven generations of children. Given that it is a relational, revitalizing praxis, “*Etuaptmumk*”—“Two-Eyed Seeing” could fundamentally change the ways in which ERH scholarship, practice, research, and advocacy operate by facilitating the co-creation of ethical spaces for co-production of new knowledge. It can be an approach to inclusive, equitable, and trustworthy collaborations with diverse families and communities [9,33,37,53,54,55,56,57]. “*Etuaptmumk*”—“Two-Eyed Seeing”, “*M’sɨt No’kmaq*”—“All our relations,” and shared healing stories can be brought forward to revitalize teaching and learning about ERH in early childhood education, social sciences, pediatric, family, and public health certificate and degree programs [33,53]. 

### 4.2. Relationships Are the Context within Which Children Learn

Across cultures, Indigenous and non-Indigenous research and wisdom converge to support a fundamental tenet of ERH, that relationships are the context within which children learn and grow [3,4,5,34,35,36,37,38,58,59,60,61,62,63,64,65]. ERH also builds on observation, reflection, dialogue, and research in child development with relationships being key to RH, development, and early learning [3,4,5,66,67,68,69,70].

### 4.3. Attachment Theory

In 2002, the American Psychological Association identified the one hundred most eminent and influential psychologists of the twentieth century [71]. All were European or American. Five were women. Two were the early attachment theorists, John Bowlby (British) and Mary Ainsworth (American), who suggested that attachment serves to keep infants close to mothers, thus improving chances of survival, and that the effects of secure and insecure types of mother–infant attachments continue throughout life [72,73,74]. Ainsworth went on to develop the “Strange Situation” protocol for discerning secure and insecure attachment patterns [74]. Critics argue that attachment theory is overly focused on the mother–child relationship and does not account for other relational, social, ecological, or cultural factors that shape infant, child, and family wellbeing [27,67,75,76,77]. Nonetheless, attachment theory inspired copious studies on the impacts of early attachment styles on child development, adaptation, parenting, maternal and infant mental health, and interpersonal relationships throughout life, as well as the development of assessment tools, inventories, classification systems, diagnostic codes, and interventions. *Circle of Security*, one of the most accessible approaches to helping families, clinicians, educators, and childcare providers understand and support children’s attachment needs, rose from years of subsequent clinical and research work [41,64,78,79,80,81,82,83,84,85]. 

Questions arose about the universality of attachment theory and whether attachment patterns differ across cultural contexts. In recent years, in the US, practitioners and researchers of Color call for movement toward antiracist perspectives in attachment theory, research, and practice [77]. Posada et al. [75] tested Bowlby and Ainsworth’s hypothesis that secure base phenomena are universal by observing mother–child dyads in nine countries on four continents. They found children did indeed use their mothers as a secure bases, with striking similarities and important, nuanced cultural differences. They did not observe how secure base and safe haven phenomena manifest in multi-member relationships and other influences on security and attachment. The Posada et al. [75] study provides evidence that culture shapes how young children and families negotiate attachment. 

Indigenous scholars challenge dominant views of attachment [67,76]. Root [76] applies “*Etuaptmumk”*—"Two-Eyed Seeing” and *M’sɨt No’kmaq* (a philosophy that maintains that everything and everyone is interconnected, has a purpose, and is worthy of respect) to an analysis of the difficulties that Bowlby- and Ainsworth-informed attachment theories and practices pose for Indigenous People. She proposes an alternative framework for understanding and supporting security, social, emotional, and relational wellbeing [8,76]. Root and Nahwegahbow’s work [36,76] enrich the understanding of children’s social emotional attachment needs. They describe efficacious approaches to raising secure, thriving children in diverse societies where caregivers draw on alternate knowledge systems and hold infants and children in multi-member relationships. Lessons learned from these and other studies [67,86] help decolonize childrearing, center traditional wisdom, and enable Indigenous communities to generate frameworks that are appropriate and desirable in their traditions. 

**Figure 1:**
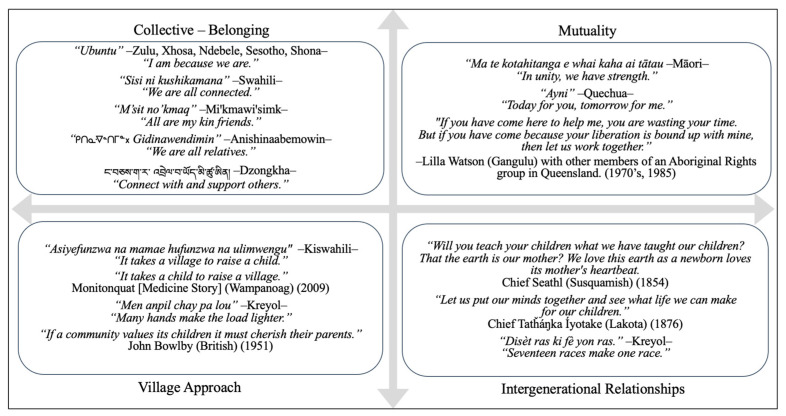
Knowledge about Relational Health (RH) exists around the world [6].

Around the globe, research by Indigenous, non-Indigenous, and cross-cultural teams of scientists demonstrate ordinary, reciprocal ways of being with infants and young children nurture relationships, security, belonging, social and emotional wellbeing, health, and learning [4,5,23,27,29,30,31,32,34,35,36,37,38,60,65,68,70,87,88,89,90,91]. Although manifestations of RH are linked to cultural context, some themes are apparent across cultures and races. Themes that underscore the relevance of key tenets of ERH—the security of knowing one belongs and is cherished, mutuality, intergenerational responsibility for the transmission of wellbeing, and the value of collective, village approaches to rearing flourishing children—are offered in Figure 1. These are presented in the spirit of “*Etuaptmumk*”—“Two-Eyed Seeing”, language justice, and inclusivity, not universality. 

The importance of connection is a tacit feature in all the teachings in Figure 1. The arrows in the matrix signify interconnections between ancestors and future generations, Mother Earth, and all living things to the east, south, west, north, and heavenward. Teachings are grouped thematically, meaning, for example, that despite distance in time, space, and cultural contexts, *belonging* is a central feature of the traditional teachings of the People of Haiti; the Mi’kmaq on the Gaspésie and in Canada’s eastern maritime provinces; the Anishinaabeg from the Ottawa River Valley west across Northern Ontario to the plains of Saskatchewan, south to the Dakotas and east to the Great Lakes; diverse ethnic groups in Africa; and the Ngalop Dzongkha-speaking people of Bhutan. 

Similarly, despite great distance in time, space, and cultural contexts, Māori children in New Zealand; Gangulu Aboriginal children and activists in Australia; and Andean children who grow up speaking Quechua (the ancient Incan language that survived European colonization) all learn the importance of *mutuality in relationships*. Respected Haitian, Susquamish, and Lakota Elders’ pass forward teachings about *intergenerational relationships and responsibilities*. There is truth in Kiswahili, Wampanoag, Haitian, and British teachings about *village approaches* to promoting and sustaining community wellbeing. 

## 5. Cultural Context: It Takes a Village—And Humility

Despite being a fundamental aspect of human experience, culture is a term that conjures deep complexity [92]. It encompasses the historic and contemporary beliefs, values, traditions, attitudes, behaviors, norms, language, customs, and practices that are passed from generation to generation [93], often without explanation, an “unconscious transmission of adaptive childrearing mechanisms” [94] (p. 103). Petty and Leach remind us that cultural practices can set societies apart and be exchanged between individuals [95].

Frameworks for family RH, parenting, child development, and ERH are deeply contextual [2,26,27,29,31,57,86,96,97,98]. A fair understanding of the development of ERH in a particular family depends on understanding their particular cultural context. Family culture is shaped by the sociocultural [96,97,98] and ecological contexts in which they live [87]. Culture influences how families perceive and engage in parenting practices; interact with each other; socialize their infants and young children; and support child development [2,3,29,30,31]. Practitioners working with families come with intersecting, implicit personal and disciplinary biases that must be recognized, mitigated and adapted to different cultural contexts [99,100]. The cultural iceberg [101,102] is often used to summarize and illuminate the complexity of culture in terms of food, language, holidays, crafts, literature, music, and more. 

Sociocultural context includes the societal structures, systems, beliefs, ideologies, attitudes, and experiences of historical and cultural trauma that exist within a society [103,104]. Social order is organized around rank, race, ethnicity, socioeconomic status, citizenship status, gender, age, politics, religion, ideological affiliations, and other aspects of identity [103]. Identities intersect [105,106]. Systems and institutions encode reminders of social order rooted in privilege, discrimination, racism, genocide, and other systems of oppression, to name a few. Reminders of the nuances of social order are transmitted via interpersonal interactions shaped by biases embedded in sociocultural contexts [103]. Context matters [87].

Cultural humility emphasizes self-reflection and openness to lifelong learning, dialogue, and mutual understanding [25]. It is compassionate curiosity—the “humbleness of not knowing” [24]—and positioning oneself at the feet of the storytellers [45,46] and is essential in cross-cultural interactions [107]. Cultural humility denotes an approach grounded in respect, empathy, and responsiveness [25]. It fosters possibility. It is necessary for openness to and mutual understanding of signs of emotionality and ERH. It enables the development of shared language, and mutual reflection on relational strengths, struggles, and circumstances that threaten wellbeing.

Strengthening ERH starts with the recognition that infants, young children, and families need a sense of belonging, positive multi-member relationships, and time to be with the people who care for them. The relationship between ERH and collective wellbeing may best be understood as a parallel process to the wellbeing of infants and “villagers” [39]. Villages that support healthy, adaptive, multigenerational social networks support ERH. 

The village approach [39] to supporting flourishing ERH ecosystems is attentive to physical environments in geographical neighborhoods where children and families live. Village members form multi-generational connections around shared languages, forms of worship, origin, sovereignty, gender, and other identities. A shared sense of belonging, roles, and responsibilities mitigate social isolation and affirm social and cultural identities that wrap around, protect and nurture the youngest village members. Ideally, the village is an “environment where children’s voices are taken seriously, where multiple people, the villagers, including parents, siblings, extended family members, neighbors, child and healthcare providers, and other community members are essential in the lives of infants, young children, and their families” [39] (p. 2). The village becomes an environment where ERH flourishes. Outsiders’ demeanor, and the extent to which they practice cultural humility and are genuinely committed to anti-colonizing practices [25,86,108,109,110,111,112] will affect efforts to join with village members for the purposes of understanding and supporting ERH. 

## 6. A Holistic Perspective: Factors That Impact ERH

Holistic refers to an approach or perspective that considers the whole person, system, or situation, taking into account all aspects of its complexity, interconnectedness, and context [12]. Holistic approaches recognize that individuals and phenomena cannot be fully understood or addressed by focusing solely on isolated parts or symptoms; rather, they emphasize the integration of physical, mental, emotional, social, cultural, and spiritual dimensions to promote overall wellbeing and wholeness. By considering all dimensions of human experience and context, holistic perspectives aim to promote health, wellness, and flourishing across diverse populations and settings.

A holistic approach is relevant when communities begin addressing structural and systemic factors beyond families’ and villagers’ control that impact their social determinants of health: economic stability, access to quality education and healthcare, access to nutritious and affordable food options, quality neighborhoods, built environments, and social communities. Systemic factors like racism, gender biases, and politics that determine their access to resources cause health disparities [113]. The Automatic Benefit for Children (ABC) Coalition is an example of a holistic approach to advancing economic and racial justice by providing a universal guaranteed income for children in the US [114]. 

Traditionally, physical health and development have been the focus of pediatric well-child visits in the US. In 2021, there was a notable change in perspective as the American Academy of Pediatrics (AAP) introduced a policy statement advocating for the promotion of ERH as a proactive strategy focusing on strengths rather than deficits [115]. “Driving this transformation are advances in developmental sciences as they inform a deeper understanding of how early life experiences, both nurturing and adverse, are biologically embedded and influence outcomes in health, education, and economic stability across the life span” [115] (p. 1). The power of relational health is that it not only buffers adversity but also promotes capacities that enable children to readily adjust to changing circumstances in the future. Yet, pedagogical and training biases, such as systems that require diagnostic labels for tracking and payment, are deeply ingrained and require intentional efforts to pivot perspectives and practices toward embracing a “funds of knowledge” [116] approach. Locating problems in individuals versus considering what happened to them [117,118] is the antithesis to “funds of knowledge” that recognizes and values the expertise, strengths, and resources that families and communities possess based on their cultural backgrounds, life experiences, and community contexts. From neuro-sequential and healing-centered perspectives, the root causes of social, emotional, and relational difficulties do not reside in infants, young children, and families alone but instead arise from what happened to them and their communities [118].

Instead of solely concentrating on problems and deficits, a holistic perspective emphasizes the mutual understanding of situations from infant, family, and community perspectives and collaborative responses to interconnected biological, relational, psychological, economic, political, and environmental factors that affect their ability to thrive [11]. Communities with stable employment, affordable housing, and equitable access to services can sustain nurturing and supportive environments for children. Over time, the relationship between RH and collective wellbeing operates in a feedback loop, with each influencing and reinforcing the other. 

In urbanized areas, where neighborhoods are increasingly diverse, transient, disconnected, or divided [119], a holistic and village perspective to ERH presents both challenges and opportunities. Family members may be scattered, less connected to social support networks, and grieving a diminished sense of community. While cultural diversity enriches communities, it can also present challenges in terms of communication and collective efforts to address the diverse needs of people from different backgrounds in an urban area [120,121]. Social isolation and loneliness are prominent effects of urbanization, particularly among people who are most impacted by systemic and structural inequities. Social isolation adversely affects parental mental health [54,122], parenting practices [123] RH, sense of belonging, hope, and possibility [124]. Burke Harris [125,126,127] described how toxic stress, the chronic stress of poverty, and childhood adversity can harm children particularly in urban areas with fragmented and inadequate services. “All of the research on ACEs is telling us that relationships are healing” [128]. Perry writes, “The key to having many healthy relationships in your life is having a few safe, stable, and nurturing relationships in your first year. This lets you get adequate repetitions to build the foundation—the fundamental relational architecture—that will allow you to continue to grow additional healthy relational connections” [118] (p. 64). 

Despite the hardships that urbanization can present, for some families in urban communities, neighborhood villages represent the possibility of community that extends beyond physical proximity. With the advent of technology and social media, virtual villages are created based on shared interests, identities, or experiences. Grassroots initiatives, community organizing, and collective action often serve to promote community engagement and collaboration, and create supportive environments that strengthen social bonds and enhance RH. Moreover, urban environments offer opportunities for cultural exchange, learning, and collaboration across diverse communities. They allow for intercultural dialogue and opportunities to witness and enrich children’s experiences, broaden their perspectives, and foster mutual understanding and respect among families from different cultural backgrounds. Practices that support ERH include informal and organized community gatherings where people of all ages can enjoy being together. 

## 7. Changing Discourses, Improving Outcomes 

Infants are meant to be held in safe, stable, and nurturing relationships (SSNR) [12]. Garner noted, “There are three foundational but often overlooked characteristics buried within this concept… Safety is more than just freedom from violence—it is also trust. Stable relationships demand repair and the ability to be kind rather than right. Nurturing is setting limits and scaffolding skills through the use of an optimistic growth mindset. Trust, repair, and an optimistic growth mindset are foundational to both ERH and the healthy cultural norms that define the way we interact with each other” [129].

ERH cannot thrive without addressing deep-rooted systemic racism in the US and the colossal impacts of colonization and crimes against humanity on bodies, relationships, bodies of land, and the villages of enslaved, displaced, and oppressed people everywhere. “Decolonization and its aims —justice, revitalization and renaissance— are work that belongs to all people, everywhere” [108], because the social constructs, theories and worldviews of colonizers become deeply embedded in the minds, bodies, relationships, and systems of subjugated people. To this point, discourses that characterize Diversity-Informed Tenets for Work with Infants, Children and Families (The Tenets) [104,130] and the three approaches to revitalizing ERH work described below resist narratives, policies, and practices that threaten to perpetuate colonialism. 

Pathways to intergenerational healing, reclaiming culture, restoring wellbeing in relationships, and reimagining thriving are needed. Ginwright [47,131] offers a framework for pivoting towards “reimagining justice, reimagining ourselves.” People who commit to ERH work resist colonialism by sustaining personal commitments to growing awareness; learning to pause, breathe, and shift from hustle toward presence and flow; intentionally pivoting from transactional to transformative relationships; and focusing on creating possibilities [47]. Resistance, healing, and revitalization require routine engagement in restorative experiences; seeding and nurturing relationships; and cultivating humility and compassionate curiosity. Augmenting soil for ERH support for families and villages involves noticing remnants of colonialism in ERH promotion and intervention efforts; taking collaborative steps towards addressing social determinants of health [132]; and leaning into diversity-informed discourse and practices [104,130] at small-group, organizational, and regional levels. 

Diversity-Informed Tenets for Work Infants, Children and Families [104,130] offer an aspirational framework for integrating equitable and inclusive practices in relational work with infants, young children, families, and their villages. Relationships, community, and contexts are bidirectional, dynamic systems that unfold in interactions, as does the work of deep equity and diversity-informed ERH. The Tenets are grounded in intersectionality theory [104], which argues that multiple systems of oppression collectively affect us all; postcolonial theory [133]; critical pedagogy [134]; and liberation psychology [135]. These call attention to the reality of indoctrination into historical and sociopolitical contexts such that the interests of particular groups are furthered at the expense of others [104,136]. 

Discriminatory policies and practices harm adults and the infants and children in their care. The Tenets are intended to promote equity and mitigate racism, implicit bias, and structural and systemic forms of oppression. Tenet 1 (*Self-Awareness Leads to Better Services for Families*) describes how individuals, organizations, and systems deepen their awareness of the impacts of implicit bias and oppression; practice compassionate curiosity; promote equity; and provide culturally attuned services. Tenet 3 (*Work to Acknowledge Privilege and Combat Discrimination*) describes how practitioners can acknowledge and use privilege strategically and responsibly to promote social justice and relationships that foster learning and healing. Tenet 4 (*Recognizing and Respecting Non-Dominant Bodies of Knowledge*) explains how and why diversity-informed practice recognizes nondominant ways of knowing, bodies of knowledge, sources of strength, and routes to healing within all families and communities and offers a tangible beacon of practice that moves us away from centering WEIRD (Western, Educated, Industrialized, Rich, and Democratic) [137] discourses, science, and policies toward centering the knowledge of Indigenous traditions and cultures and people who are marginalized in our communities and countries. 

Three approaches to revitalizing ERH work were selected to exemplify the *what* (content), *how* (approach), and *who* (the people) aspects of inclusion-, equity-, and diversity-informed ERH discourse and practice. The selection criteria were as follows: 1. Open pathways to RH by centering families and relationships with infants and children; 2. Be inclusive in approaches to engaging and supporting families in terms of materials, video, stories, and simple illustrations, without requiring access to technology beyond a smart phone or high levels of literacy; 3. Facilitate oral storytelling and mutual reflection; 4. Offer opportunities for caregivers to hear, share, and reflect on real-life situations they face with their babies within their particular cultural contexts; 5. Make the content, insights, aspirations, ideas, and solutions that caregivers and children offer as relevant as those that practitioners offer so together they can engage in dialogue, mutual reflection, and action.

*Small Moments, Big Impact* (SMBI) [138]. Because the pediatric visit in the US is where more than three-quarters of children are seen, conversations about ERH during these encounters opens the door for universal promotion and intervention. SMBI is a way pediatricians can center RH during brief encounters with patients and their families. It offers a rich, easily accessible online library of videos of racially, culturally, and linguistically diverse families interacting with their infants and explaining stressful and heartening experiences during the first six months of life after birth, plus informative videos of a pediatrician speaking directly to them about expectations before birth and during the months that follow. Guidance is offered to users about being fully present; listening sensitively to families’ stories and reflections; and co-developing mutual plans for next steps. In a small pilot with mothers, Zuckerman and colleagues [138] found that SMBI participants reported more positive child-rearing beliefs and efforts to understand their children’s feelings than control group members.

*Simple Interactions* (SI) [2,65,139]. SI offers common, descriptive language and simple drawings that can be used to describe interactions and relationships between caregivers and multiple children in inclusive care settings, with children of different ages, abilities and ways of engaging and responding to others. These invite shared observation, commentary, and reflection on and about brief recordings of interactions between adults and children. In practice, this means observing and capturing ordinary moments in authentic environments, thus inviting dialogue among proximity experts (child helpers themselves). Best practices arise from noticing what adults and children already do well together. In this way, SI offers members of a village a collaborative way to develop and advance a shared vision for their children. SI is committed to promoting human development and empowering adult helpers and local communities across many states and several countries. 

*Early Relational Health Conversations* (ERH-C) [45,46]. ERH-C is a family reflection model for revitalizing ERH promotion and intervention. Africentric values of interdependence and collective wellbeing underlie the model’s eight components. ERH-C practitioners see interactions between infants and family members as co-created, extemporaneous narratives. Very little structure is imposed on family–baby interactions during an ERH-C visit, so the brief narratives are unscripted. Foregrounding families’ cultural contexts enables a shared, strengths-based understanding of relationships within the family. Revitalizing Reflective Consultation (R-RC) is a unique component of the model because self-awareness and self-efficacy [140] can be indirect influences on health. R-RC focuses attention on parallel processes, interpersonal, and structural factors that influence RH. R-RC is a way to monitor the relevance and utility of ERH-focused visits for families and phenomena that occur during visits. Studies are needed that examine assumptions embedded in ERH-C, the model’s relevance and utility, and funding for community-based efforts to better support infants and families in diverse villages globally.

## 8. Discussion

Cultural context has a bearing on the ways in which people understand ERH and the meanings they make of behaviors they witness during infant–family interactions. ERH ever more exposes the reality of persistent deep-rooted cultural biases, systemic and structural racism [141,142] that do not serve infants and families well. Racism is so embedded in systems that such policies and practices are generally assumed to reflect the natural, inevitable order of things [140]. To these points, revitalizing ERH includes addressing implicit biases and “established beliefs and attitudes that produce, condone, and perpetuate widespread unfair treatment of people of color” [140] (p. 171).

During an interview for *Nurture Connections Newsletter* in 2023, Garner [49,89,129,143] explained how ERH principles will impact his work and the field of Pediatrics at large: “Most pediatricians [and other practitioners] know in their hearts that ERH is absolutely foundational to all that they do every day. Yet everyday they are actively discouraged from embracing these strength-based principles by [systems that are] squarely deficits-based.” Garner went on to say, “The entire healthcare–insurance–pharmaco–industrial complex runs on two codes. A diagnosis code that defines what is wrong with the patient, and a procedural code that defines what was done to the patient.” Constrictions on time, space, funding, and reimbursement for strength-based, revitalizing services like ERH impact doulas, Infant and Early Childhood Mental Health Consultants (IECMHC), Early Intervention (EI) providers, community health workers, and childcare providers as well. Furthermore, Indigenous, African American, Asian American, and Latinx people have good reason to fear relationship-focused interventions in education, child welfare, and criminal justice systems that are similarly deficit-based, defined by oppressive procedural codes, rife with racism, and histories of oppression and genocide [144,145,146,147]. Braveman et al. [140] explain causal pathways to health harms and offer strategies to identify and address inequities in the key determinants of health, such as economic security, housing security, educational opportunity, and treatment by child welfare and criminal justice systems. 

Garner concluded, “The cognitive dissonance between what pediatricians [and other practitioners] know in their hearts and actually do to keep the lights on [in their workplaces the US] is simply disorientating.” A conference featuring “*Etuaptmumk”*—"Two-Eyed Seeing” approaches [8] to addressing inequities in the key determinants of health [132], mitigating and lifting up ERH around the world could inspire much-needed creativity and innovation. 

Meanwhile, concrete steps that service providers and agencies can take to advance revitalization and support safe, stable, and nurturing relationships include advocating for changes in workflow and funding so that childcare providers, infant and early childhood educators, community health workers, and pediatricians can participate in monthly R-RC. Providers’ engagement in quality, monthly R-RC unmasks biases that perpetuate colonialism and makes empowering experiences during dialogues about ERH possible for families. When families, kin, childcare providers, and village members participate in and contribute to revitalizing practices, intergenerational healing, health and positive developmental outcomes become possible for infants and the people who care for them. Something meaningful and memorable happens for people who care for infants when the following five experiences co-occur: (1) they co-create partnerships with people who support them in taking a reflective, culturally contextualized look at relationships with their infants; (2) they see themselves and their infants as storytellers; (3) they have an opportunity to watch their stories and notice and talk about moments of connection that stand out to them and seem to stand out to their infants; (4) they recognize mutual capacities that can help them traverse adversity and thrive; and (5) they themselves have safe, stable, and nurturing relationships with their care providers. In this way, infant-by-infant, enduring, revitalizing ERH narratives are created and sustained.

## 9. Conclusions and Future Directions

ERH is a quality of relationships co-created by infants, kin, and villagers from pregnancy through childhood. Interactions can be understood as rhythms, dances, or extemporaneous stories. Relationships themselves are not ERH; rather, ERH can be a feature of relationships. Mutual capacities in a relationship have potential to hearten everyone in the relationship. The early recognition of relational health and vulnerability is important because ERH is a central factor in child health, development, and emotional and social wellbeing. ERH may also affect family wellbeing, hence highlighting the importance of infant and family-centered care. Across cultures, there are differences in the ways that overarching emotionality and mutual capacities are expressed, understood, and valued, hence exemplifying the importance of centering cultural context and families’ perspectives. 

Further study is needed to understand how RH is expressed, understood, and supported in diverse cultures across the globe. The application of “*Etuaptmumk”*—"Two-Eyed Seeing” and the *Indigenist Ecological Systems Model* [57] to ERH research and practice would be a valuable contribution to the literature. Indigenous knowledge systems-informed, community-based participatory action research and crosswalks between ERH constructs and villages’ cultural contexts will be key to adapting and scaling up ERH promotion and intervention efforts globally. Inclusive and equitable funding for village-led projects to illustrate and describe cultural context, mutual capacities, and other indicators of RH in multimember relationships would broaden and deepen the understanding of the expression and development of ERH [57,67].

Racism, colonialism, systemic barriers, and disparities impede access to information, resources, and services for too many children and families. Revitalization, Diversity-Informed Tenets, deep equity frameworks, holism, cultural humility, compassionate curiosity, and “Two-Eyed Seeing” speak to how providers, researchers, and communities are called to engage with families in support of ERH. 

Scaling up ERH also involves images, language, and messaging that is non-stigmatizing, easily accessible, relevant, and useful to racially and culturally diverse fathers, gender-diverse families, siblings, and other caregivers, as well as mothers. All families need non-stigmatizing, easily accessible paths to quality supports for ERH. As the Diversity-Informed Tenets remind us, inclusive and equitable resource allocation and leadership are needed to ensure that children and families have equitable access to the social determinants of health: family economic stability; food security; housing security in safe and healthy neighborhoods; access to quality healthcare and education; and positive connections with community. 

You cannot claim to care about ERH while ignoring the impact of structural racism and economic disparities. Mobilization around RH could help communities pivot from transactional relationships to transformative relationships [47] and cultivate deeper human connections that advance restoration and healing. “Critical reflexivity is more than individual self-awareness. It also requires that we constantly evaluate ways in which we contribute to liberation and oppression” [86] (p. 211) for we are all owners of humanity. 

## Figures and Tables

**Table 1:** Indicators of ERH when overarching emotionality in relationships is positive [6].

▪ The mother or birthing person (BP) and others acknowledge the infant’s presence and movements in the womb. ▪ The infant may move or still in response to stimuli outside the womb. ▪ Kin and friends tend to the safety and security of the BP and infant. ▪ Kin and friends express acceptance, wonder, or delight in the pregnancy, the BP, or unborn child.
▪ The newborn orients toward the BP and is welcomed by them. ▪ The newborn settles against the BP’s chest where heartbeat and scent are readily perceptible. Both are calm. BP feels relaxed and comfortable. ▪ The newborn orients toward voices of people who spoke to it often while in utero. They notice and respond positively. ▪ The newborn, BP, a sibling, or other person in close relationship to the infant gaze into each other’s eyes during the newborn’s quiet alert states. They may breathe in one another’s scent.
▪ When the infant is upset, those in close relationship use themselves successfully to soothe the infant, instead of leaving the infant alone, or alone with an object. ▪ Mutual calm interest in one another. ▪ When the infant gives open mouth kisses to a person in close relationship, they interpret it as affection and respond with affection—a sign of mutual intimacy. ▪ Mutual engagement, responsiveness, attention, and enjoyment. ▪ Mutual inclusion of another person in a three- or four-way interaction.
▪ When other(s) in relationship with the infant mimic its sounds, gestures, and other movements, the infant repeats them with pleasure. Turn-taking may become a mutually delightful game. ▪ Mutual pleasure in reciprocal communication and actions—not necessarily with words. ▪ Mutual ability to initiate and follow one another’s lead. ▪ Shared humor. ▪ Mutual cooperation during daily routines.
▪ Mutual ability and willingness to work through small challenges. ▪ Joint storytelling. ▪ Mutual exploration of emotions including frustration, joy, fear, and sadness. ▪ Mutual engagement in conversations about connections between their own and others’ intents, actions, and desirable and undesirable effects.

## Data Availability

No new data were created or analyzed for this essay. Data sharing is not applicable to this article.
